# Rapid Resolution of Cholangitic Abscess and Biliary Sepsis in a Liver Transplant Recipient after Hepatic Artery Revascularization

**DOI:** 10.1155/2016/1520849

**Published:** 2016-06-01

**Authors:** Sushrut Trakroo, Kamran Qureshi

**Affiliations:** ^1^Department of Surgery, Temple University Hospital, Philadelphia, PA 19140, USA; ^2^Section of Gastroenterology and Hepatology, Temple University Lewis Katz School of Medicine, Philadelphia, PA 19140, USA

## Abstract

Hepatic arterial flow is paramount in preserving biliary integrity. We present an interesting clinical scenario of a liver transplant recipient with biliary anastomotic stricture who developed biliary abscess and sepsis after Endoscopic Retrograde Cholangiopancreatography. The abscess did not respond to maximal medical management, percutaneous drainage, and adequate endoscopic biliary drainage. Clinically, patient continued to deteriorate and imaging identified hepatic artery stenosis which was treated with percutaneous intra-arterial stenting. Revascularization and perfusion of infected area led to rapid resolution of abscess and sepsis. This case emphasizes the anatomic basis of biliary ductal pathology. An important educational point is to understand that interrupted hepatic arterial supply can lead to biliary complications in liver transplant recipients and early correction of perfusion deficit should be pursued in such cases. In nonresolving hepatobiliary infections after liver transplantation, hepatic arterial compromise should be looked for and if present promptly treated. Reperfusion of biliary system in our patient led to improved antibiotics penetration, resolution of abscess and sepsis, and healing of biliary stricture.

## 1. Introduction

Branches of hepatic artery and arterioles supply the arterial blood to whole of biliary tree via peribiliary plexus of capillaries and then drain into hepatic sinusoids. Incidence of hepatic artery stenosis (HAS) after liver transplant (LT) is considered to be 4 to 10% after deceased donor LT [1, 2]. Presentation of delayed HAS is less clear, ranging from asymptomatic to progressive biliary disease in the form of strictures [3]. Studies have shown decreased incidence of biliary pathology with early intervention [4].

## 2. Case Report

**Figure 1:**
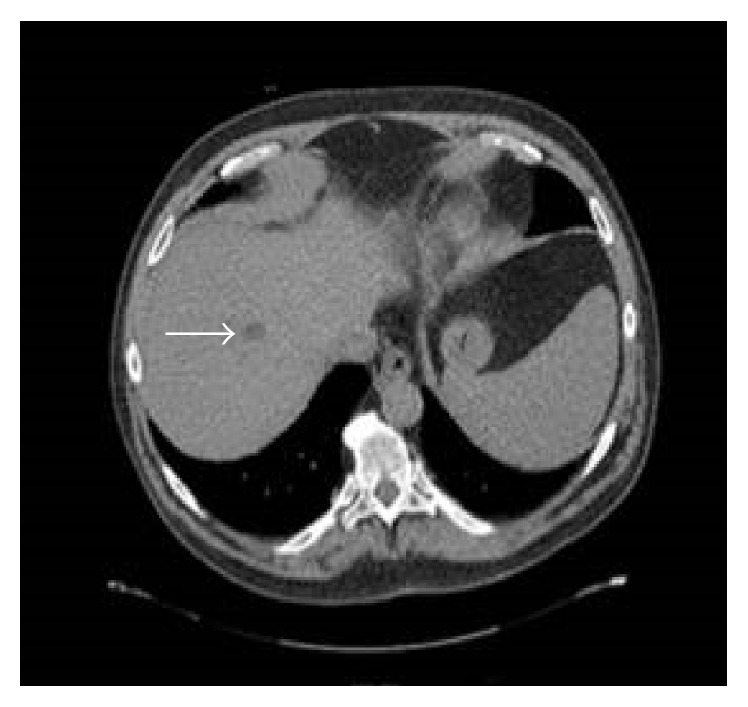
Multidetector CT of the abdomen on the day of admission showed a new ill-defined hypodense lesion in the right hepatic lobe.

**Figure 2:**
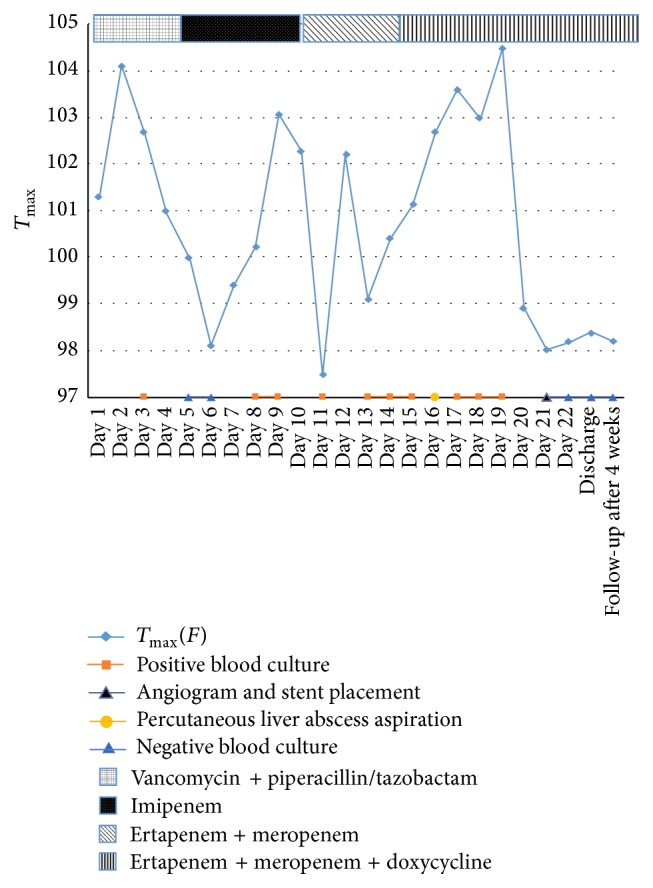
Timeline graph to depict various clinical parameters during the admission and after discharge from hospital.

**Figure 3:**
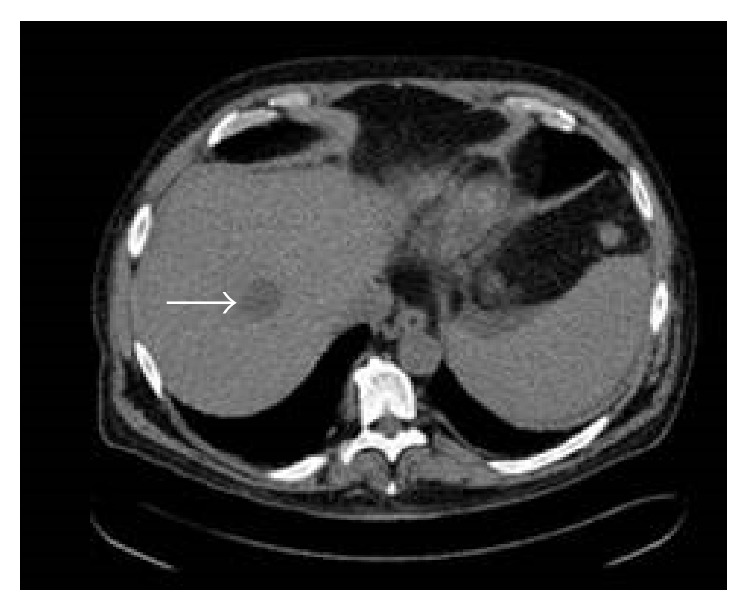
CT image on day 14 redemonstrated enlarged ill-defined hypodensity within the right hepatic lobe.

**Figure 4:**
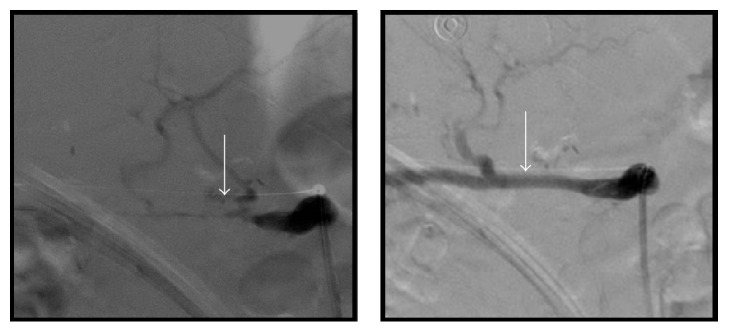
Angiogram with hepatic artery stent placement on day 21 showed critical stenosis of the anastomotic segment of replaced hepatic artery. Subsequent balloon dilatation and stenting with 2–5 mm stents was completed.

**Figure 5:**
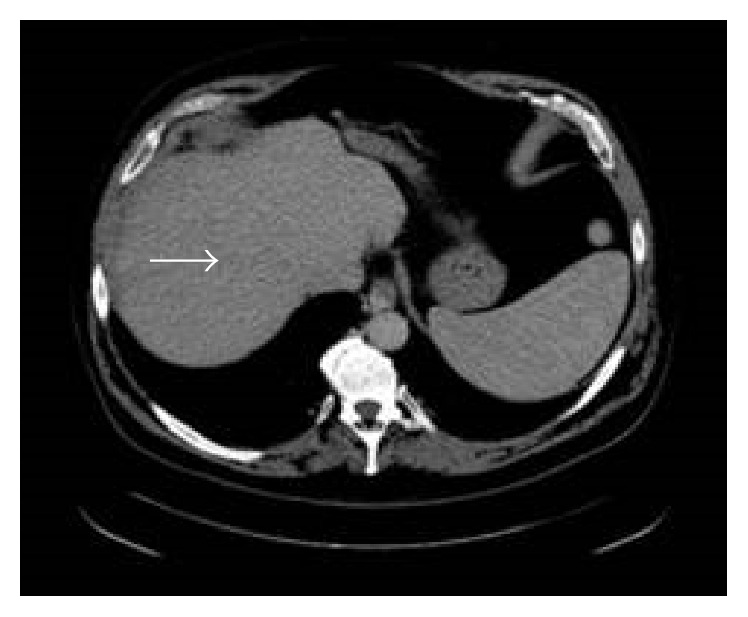
Follow-up CT image at 3 months after hepatic artery revascularization with stent placement showed resolution of liver abscess.

This sixty-year-old male with a history of deceased donor LT done for cryptogenic liver cirrhosis over 3 years ago presented to the emergency department with 3 days of high grade fevers and chills. He was found to be tachycardiac and hypotensive and thus was admitted to the Intensive Care Unit (ICU) for management of septic shock. Review of his past history revealed uneventful 2.5 years after LT course and a diagnosis of late onset biliary anastomotic stricture, which was recognized during the work-up of new onset cholestatic hepatitis 6 months prior to this admission. During the original LT operation, surgeons had to perform hepatic arterial reconstruction because of donor to recipient arterial size mismatch. For the treatment of biliary anastomotic stricture he underwent Endoscopic Retrograde Cholangiopancreatography (ERCP) with biliary stent placement 3 times during the course of subsequent 6 months. His last ERCP procedure was performed 7 days prior to this ICU admission; during which a severe localized biliary stricture was found and was treated with balloon dilation and exchange of biliary stents. Four days after the ERCP he started mounting high grade fevers with chills, which brought him to the hospital. Clinical presentation on admission (day 0) suggested presence of septic shock with right upper abdominal pain and new onset jaundice. After initial fluid resuscitation, an abdominal computerized tomography (CT) scan was done which showed a new liver lesion (Figure 1) thought to be abscess in this clinical scenario. He was managed for septic shock secondary to a working diagnosis of ascending cholangitis/hepatic abscess and was started on broad-spectrum antibiotics (vancomycin with piperacillin/tazobactam), along with vasopressor support. Blood cultures (BC) from admission eventually grew extended spectrum beta-lactamase producing (ESBL)* Klebsiella pneumoniae* and antibiotic coverage was accordingly modified to imipenem/cilastatin. With an adequate antibacterial coverage and following initial response to imipenem/cliastatin regimen (and negative BC), he started to develop fevers again. Repeat BC (day 8) showed the same bacterial growth of ESBL* Klebsiella pneumonia* (Figure 2). Subsequently antibiotic coverage was broadened with double carbapenem regimen (ertapenem with meropenem) for presumed carbapenemase producing pandrug resistant* Klebsiella pneumonia*. Repeat CT scan showed enlarging liver abscess (Figure 3), as he continued to have fever and bacteremia despite the addition of doxycycline in the antimicrobial regimen. Thus, CT guided percutaneous drainage of abscess was performed (day 16) and aspirate grew the same ESBL* Klebsiella pneumonia*, which was persistently seen in the blood stream. Despite aggressive medical management for total of 3 weeks, he remained febrile with bacteremia and sepsis. HAS been suspected and was identified on ultrasound arterial Doppler study. Subsequently, he underwent hepatic artery angiogram (day 21), which showed critical anastomotic stenosis of the replaced common hepatic artery that arose directly off the aorta. Nitroglycerin (200 mcg) was administered for vasodilatation and mechanical dilatation of the stricture was performed with a 4 mm balloon dilatation catheter across the stenotic segment. A 5 mm × 15 mm express SD stent was then deployed across the stenotic segment. Completion angiogram demonstrated excellent perfusion to the liver (Figure 4). The revascularization resulted in reperfusion of the biliary system and better access and penetration of antibiotics to the infected area and abscess. He showed dramatic clinical and hemodynamic improvement over the subsequent 24 hours. Blood cultures drawn the day after the arterial stenting showed no growth and he was discharged 3 days after revascularization along with the confirmation of sterile BC. 3 months follow-up CT scan (Figure 5) showed resolution of liver abscess and ERCP done afterwards showed a resolved biliary anastomotic stricture.

## 3. Discussion

Hepatic arterial flow is paramount in preserving biliary integrity [5, 6]. Delayed presentation of HAS after LT is rare and can lead to potential graft loss and mortality [7]. Pathophysiology of delayed HAS includes transplant associated arteriosclerosis, vascular intimal thickening, medial attenuation, and vasoconstriction [8, 9]. Our patient's clinical condition deteriorated despite maximal medical management and percutaneous abscess drainage. This can be explained on the basis of impaired perfusion of the affected/infected area which limited antibiotic penetration. Recognition and stenting of HAS led not only to immediate resolution of sepsis and abscess but also to the resolution of biliary stricture. He did not require any further interventions for biliary stricture on the follow-up ERCP done 3 months later.
